# Concordance of regional hypoperfusion by pCASL MRI and ^15^O-water PET in frontotemporal dementia: Is pCASL an efficacious alternative?

**DOI:** 10.1016/j.nicl.2022.102950

**Published:** 2022-01-31

**Authors:** Tracy Ssali, Lucas Narciso, Justin Hicks, Linshan Liu, Sarah Jesso, Lauryn Richardson, Matthias Günther, Simon Konstandin, Klaus Eickel, Frank Prato, Udunna C. Anazodo, Elizabeth Finger, Keith St Lawrence

**Affiliations:** a Lawson Health Research Institute, London, Canada; b Department of Medical Biophysics, Western University, London, Canada; c St. Joseph’s Health Care, London, Canada; d Fraunhofer Institute for Medical Image Computing MEVIS, Bremen, Germany; e University Bremen, Bremen, Germany; f Mediri GmbH, Heidelberg, Germany; g Department of Clinical Neurological Sciences, Western University, London, Canada

**Keywords:** Frontotemporal dementia (FTD), Cerebral blood flow (CBF), Radiolabeled water PET (^15^O-water), Arterial spin labeling (ASL), PET/MRI, sensitivity

## Abstract

**Background:**

Clinical diagnosis of frontotemporal dementia (FTD) remains a challenge due to the overlap of symptoms among FTD subtypes and with other psychiatric disorders. Perfusion imaging by arterial spin labeling (ASL) is a promising non-invasive alternative to established PET techniques; however, its sensitivity to imaging parameters can hinder its ability to detect perfusion abnormalities.

**Purpose:**

This study evaluated the similarity of regional hypoperfusion patterns detected by ASL relative to the gold standard for imaging perfusion, PET with radiolabeled water (^15^O-water).

**Methods and materials:**

Perfusion by single-delay pseudo continuous ASL (SD-pCASL), free-lunch Hadamard encoded pCASL (FL_TE-pCASL), and ^15^O-water data were acquired on a hybrid PET/MR scanner in 13 controls and 9 FTD patients. Cerebral blood flow (CBF) by ^15^O-water was quantified by a non-invasive approach (PMRFlow). Regional hypoperfusion was determined by comparing individual patients to the control group. This was performed using absolute (aCBF) and CBF normalized to whole-brain perfusion (rCBF). Agreement was assessed based on the fraction of overlapping voxels. Sensitivity and specificity of pCASL was estimated using hypoperfused regions of interest identified by ^15^O-water.

**Results:**

Region of interest (ROI) based perfusion measured by ^15^O-water strongly correlated with SD-pCASL (R = 0.85 ± 0.1) and FL_TE-pCASL (R = 0.81 ± 0.14). Good agreement in terms of regional hypoperfusion patterns was found between ^15^O-water and SD-pCASL (sensitivity = 70%, specificity = 78%) and between ^15^O-water and FL_TE-pCASL (sensitivity = 71%, specificity = 73%). However, SD-pCASL showed greater overlap (43.4 ± 21.3%) with ^15^O-water than FL_TE-pCASL (29.9 ± 21.3%). Although aCBF and rCBF showed no significant differences regarding spatial overlap and metrics of agreement with ^15^O-water, rCBF showed considerable variability across subtypes, indicating that care must be taken when selecting a reference region.

**Conclusions:**

This study demonstrates the potential of pCASL for assessing regional hypoperfusion related to FTD and supports its use as a cost-effective alternative to PET.

## Highlights

• ASL is an alternative to ^15^O-water for identifying hypoperfusion in FTD patients.
• ROI-based perfusion by ASL and ^15^O-water were strongly correlated (R > 0.75).
• Hypoperfusion patterns identified by ^15^O-water and ASL were in good agreement.
• Careful selection of the reference region is required to avoid erroneous results.

## Table of acronyms

^15^O-water: Radiolabeled Water
^18^F-FDG: Radiolabeled Fluorodeoxyglucose
aCBF: Absolute Cerebral Blood Flow
ACE-III: Addenbrooke's cognitive examination III
AD: Alzheimer's Disease
ASL: Arterial Spin Labeling
bvFTD: Behavioural Variant of FTD
CBF: Cerebral Blood Flow
CBS: Corticobasal Syndrome
ENABLE: ENhancement of Automated Blood fLow Estimates
FBI: Frontal Behavioural Inventory
FL_TE-pCASL: Free Lunch Pseudo Continuous Arterial Spin Labeling
FMRIB: Functional Magnetic Resonance Imaging of the Brain
FNIRT: FMRIB's Nonlinear Image Registration Tool
FOV: Field of View
FSL: FMRIB's Statistical Library
FTD: Frontotemporal Dementia
GRASE: Gradient and Spin Echo
ICA: Internal Carotid Artery
LD: Labeling Duration
M0: Equilibrium Magnetization Image
MATLAB: MathWorks Laboratory
MBq: Mega Becquerel
MNI: Montreal Neurological Institute
MPRAGE: Magnetization Prepared and Gradient Echo
MRI: Magnetic Resonance Imaging
nfPPA: Nonfluent Primary Progressive Aphasia
PC: Phase Contrast
PET: Positron Emission Tomography
PLD: Post Labeling Delay
PMRFlow: Non-invasive Hybrid PET/MR Flow
PPA: Primary Progressive Aphasia
PSP: Primary Supranuclear Palsy
rCBF: Relative Cerebral Blood Flow
ROI: Region of Interest
SD-pCASL: Single Delay Pseudo Continuous Arterial Spin Labeling
SPM: Statistical Parametric Mapping
svFTD: Semantic Variant of FTD
T1: Longitudinal Relaxation Rate
TAC: Tissue Activity Curve
TE: Echo Time
TR: Repetition Time
VA: Vertebral Artery

## 1 Introduction

Frontotemporal dementia (FTD) is a heterogeneous class of syndromes characterized by progressive degeneration of the frontal and temporal lobes. Clinically, FTD is subdivided into behavioural variant (bvFTD), which presents with changes in personality; primary progressive aphasias (PPA) including the semantic variant (svFTD) and nonfluent agrammatic variant PPA (nfPPA), which present with language impairment; and the related syndromes including, corticobasal syndrome (CBS) and progressive supranuclear palsy (PSP), presenting as affected motor control and coordination (Mackenzie and Neumann, 2016). While early diagnosis is critical for timely inclusion in clinical trials, accurate differential diagnosis at the early stages remains a challenge due to the overlap of clinical symptoms not only among subtypes but also with neuropsychiatric diseases including Alzheimer’s disease and schizophrenia (Iturria-Medina et al., 2016, Mendez et al., 2007).

Functional brain imaging methods are commonly used to provide objective measures of disease progression that are more sensitive than changes in brain volume (Olm et al., 2016). Glucose metabolism by ^18^F-fluorodeoxyglucose (FDG) positron emission tomography (PET) is a well-established measure that is highly correlated with neuropsychiatric scores (Beyer et al., 2021) and used clinically for improving diagnostic confidence (Foster et al., 2007, McKhann et al., 2001). Due to the tight relationship between metabolism, blood flow, and brain activity, perfusion can also be used as a marker of brain health. The current standard for imaging perfusion is PET with radiolabeled water (^15^O-water) as it provides quantitative and stable results with a short scan period (2–5 min) (Ohta et al., 1996). Despite the demonstrated value of these PET-based techniques, PET imaging is expensive and access limited. Furthermore, perfusion imaging using ^15^O-water is challenging due to the short half-life of the tracer and additionally, quantification requires arterial sampling, which is invasive and sensitive to noise.

Arterial spin labeling (ASL) is an attractive alternative since it is totally non-invasive and quantitative. As an MRI-based technique, it is more accessible, cost-effective, and less technically demanding than PET. Furthermore, with the emergence of tracers for investigating dementia pathophysiology, including tau accumulation and neuroinflammation, implementing ASL as a marker of metabolic/perfusion deficits “frees” PET for more targeted studies (van Waarde et al., 2021). Several studies have investigated the ability of ASL to differentiate between FTD and Alzheimer’s Disease (AD) (Binnewijzend et al., 2014, Bron et al., 2016, Du et al., 2006, Hu et al., 2010, Tosun et al., 2016); however, there has been a dearth of studies assessing perfusion changes among the FTD subtypes. Expected patterns of regional hypoperfusion have only been identified in a few subtypes, namely bvFTD (Anazodo et al., 2018, Binnewijzend et al., 2014), and svFTD (Olm et al., 2016). Beyond group analysis involving patients with nfPPA (Hu et al., 2010) and FTD-related disorders including PSP and CBS (Cheng et al., 2020), to date there have been no studies assessing regional hypoperfusion associated with these subtypes.

Considering that prognosis and treatment options differ between subtypes (Tsai and Boxer, 2014), the aim of this study was to assess the ability of ASL to detect regional perfusion deficits related to FTD and related disorders. Hypoperfusion was determined using single-delay pseudo continuous ASL (SD-pCASL), as it is the recommended version for dementia studies (Alsop et al., 2015). Given that longer arterial transit times can be a significant source of error when using ASL with elderly and clinical populations (Alsop et al., 2015, Dai et al., 2017), this study also included a free lunch Hadamard time-encoded pCASL sequence (FL_TE-pCASL), which is able to generate transit-time-corrected perfusion images in a time efficient manner (Samson-himmelstjerna et al., 2016). In contrast to previous studies that compared perfusion to glucose metabolism (Anazodo et al., 2018, Ceccarini et al., 2020, Fällmar et al., 2017, Verfaillie et al., 2015), the current study is the first to perform a head-to-head comparison to ^15^O-water PET. Given that up to 61% of FTD cases have a vascular component (Hachinski et al., 2019, Toledo et al., 2013), potential differences related to perfusion-metabolism decoupling are avoided. Furthermore, by taking advantage of hybrid PET/MRI, cerebral perfusion could be imaged simultaneously by pCASL and ^15^O-water, thereby avoiding potential differences related to repositioning and physiological fluctuations. This study focused on single-subject analysis to account for the heterogeneity of perfusion deficits between FTD subtypes and to reflect the use of imaging in clinical studies.

## 2 Materials and methods

### 2.1 Study participants

Eleven patients with FTD or PSP and 13 age-matched neurologically healthy controls were enrolled between November 2019 and July 2021. The participants included in current study were part of a larger study evaluating the reproducibility and sensitivity of ASL among individuals with FTD (Ssali et al., 2021). Patients were recruited from the Cognitive Neurology and Aging Brain clinics at Parkwood Hospital (St Joseph’s Health Care London), while controls were recruited through the clinic’s volunteer pool. Diagnosis, performed by a clinical neurologist (E.F.), followed established consensus criteria for probable FTD (Gorno-Tempini et al., 2011, Rascovsky et al., 2011) or PSP (Höglinger et al., 2017) and included neuropsychological testing, clinical MRI, and genetic testing. Participants completed standardized psychometric assessments (Table 1) to evaluate domains of cognition. Patients’ study partners completed ratings of symptoms and behaviours. Exclusion criteria included any significant neurologic or psychiatric disorders other than suspected FTD, any significant systemic illness, and MRI incompatibility.

**Table 1:** Summary of demographics and scores from standardized psychometric assessments.

Demographics	Patients		Controls	
Sex (M:F)	3:6		8:5	
Age (years)	68.9 ± 8.2		64.1 ± 9.9	
Diagnosis	2 bvFTD 2 nfPPA 3 svFTD 2 PSP		–	

Cognitive Measures				

	N	Score	N	Score

**ACE-III Total Score (100)** (American Version A)	9	60 ± 16.6	13	92.2 ± 3.7*§*
Attention (18)	9	15 ± 2.2	13	16.7 ± 2
Memory (26)	9	12.6 ± 7.6	13	23.5 ± 2.8*§*
Fluency (14)	9	4.3 ± 3.2	13	11.8 ± 2.2*§*
Language (26)	9	15.8 ± 7.8	13	25.4 ± 1.1*§*
Visuospatial (16)	9	12.3 ± 2.2	13	14.8 ± 1.1*§*
**Mini-ACE Total Score (30)**	9	15 ± 6.9	13	27.7 ± 2.5*§*
**Boston Naming (15)**	9	5.9 ± 5.9	10	13.7 ± 1.6*§*
**Geriatric Depression Scale (Short Form; 15)**	9	5.1 ± 2.7	10	1.9 ± 3.7*§*

Cognitive Measures				

**Neuropsychiatric Inventory Total Score (144)**	8	14.3 ± 14.7	–	–
**FBI Total Score (72)**	9	24.3 ± 12.9	–	–
**Cornell (38)**	8	8.5 ± 5.4	–	–
**Cambridge Behavioural Inventory Revised (180)**	9	49.6 ± 19.7	–	–

Values are expressed as the mean ± standard deviation.

Values in parenthesis represent the maximum score for each test.

T-tests were conducted to test for differences in cognitive measures between patients and controls.

Statistical significance (p < 0.05) is indicated by §

The study was approved by the Western University Health Sciences Research Ethics Board and was conducted in accordance with the Declaration of Helsinki ethical standards. Participants provided written informed consent in compliance with the Tri-Council Policy Statement of Ethical Conduct for Research Involving Humans.

### 2.2 PET/MRI acquisition

PET and MRI data were acquired on a hybrid PET/MRI scanner (Siemens Biograph mMR) using a 12-channel PET-compatible head coil. Five minutes of list mode data were acquired immediately after a bolus injection of ^15^O-water through the antecubital vein (741 ± 67 MBq). PET data were reconstructed to 37 image volumes (frames: 3 s × 20, 5 s × 6, 10 s × 6, 30 s × 5, FOV: 172 × 172 × 127 mm^3^, voxel-size: 2.09 × 2.09 × 2.03 mm^3^) using a vendor-based MR attenuation correction map (Dixon plus bone (Martinez-Moller et al., 2009, Paulus et al., 2015)) and an iterative algorithm (ordinary Poisson ordered subset expectation maximization, 3 iterations, 21 subsets, 3D Gaussian filter of 4 mm) with corrections for decay, scatter, and dead time.

PMRFlow (Ssali et al., 2018) was implemented to generate quantitative perfusion images by ^15^O-water without arterial blood sampling. Briefly, whole-brain perfusion measured by phase contrast (PC) MRI was used to calibrate ^15^O-water images. The internal carotid (ICA) and vertebral arteries (VA) were identified using a 3D time-of-flight MRI angiography and the PC imaging plane was angulated perpendicularly to these vessels with a focus on optimizing the angle for the larger vessels (i.e. ICAs). Retrospectively gated PC images were acquired simultaneously to the ^15^O-water acquisition (TR/TE: 43.8/4.39 ms, voxel size: 0.7 × 0.7 × 5 mm^3^, FOV: 263 × 350 × 350 mm^3^, velocity encoding: 70 cm/s in the through plane direction, segments: 3). Twelve phases per cardiac cycle with four averages were acquired for a total scan time of ∼ 4–5 min, depending on the participants heart rate.

Within 5 min of the PET scan, SD-pCASL data were acquired with a 4-shot gradient and spin echo (3D-GRASE) readout (Günther et al., 2005); TR/TE: 4500/22.14 ms, voxel-size: 4 mm isotropic, FOV: 256 × 256 × 128 mm^3^, label-control pairs: 16, bandwidth: 2298 Hz/Px, 1 preparing scan, scan time: 9:46 min. In accordance with guidelines for imaging clinical populations, the post-labeling delay (PLD) and labeling duration (LD) were set to 2000 ms and 1800 ms, respectively (Alsop et al., 2015). To quantify perfusion in physiological units, an equilibrium magnetization (M0) with identical parameters except for a TR of 7000 ms and no background suppression or labeling was used.

FL_TE-pCASL images were acquired with a 2-shot GRASE readout with TR/TE: 5500/21.22 ms, voxel-size: 5 mm isotropic, FOV: 320 × 215 × 120 mm^3^, bandwidth: 2894 Hz/Px, slice partial Fourier: 6/8, phase partial Fourier: 6/8, 4 measurements per PLD, scan time: 5:52 min. With FL_TE-pCASL, the traditional PLD is replaced with time-encoding blocks (Samson-himmelstjerna et al., 2016, Teeuwisse et al., 2014). An N = 8 Hadamard scheme was applied with sub-bolus duration of 250 ms, free-lunch LD of 2000 ms, and PLD = 200 ms. This corresponded to PLD_1_/LD_1_: 1700 ms/2000 ms, and LD_2-7_: 250 ms, PLD_2-7_: 1450, 1200, 950, 700, 450, 200 ms. An M0 image was acquired with identical parameters except no background suppression or labeling.

For each participant, the labeling plane offset of the pCASL sequences was adjusted (90–125 mm from the center of the imaging slab) to ensure the vessels were perpendicular to the labeling plane (Dai et al., 2017). Background suppression was achieved using two inversion pulses to null components with T1 of 700 and 1400 ms (Günther et al., 2005). This was implemented in both pCASL sequences.

T1-weighted images, used for anatomical reference and to generate brain masks, were acquired using a 3-dimensional magnetization prepared rapid acquisition gradient echo (MPRAGE) sequence (TR/TE: 2000/2.98 ms, voxel size: 1 mm isotropic, field of view (FOV) 256 × 256 × 176 mm^3^, scan time: 4:38 min).

### 2.3 Image processing

Image analysis was performed with the Oxford Centre for Functional MRI of the Brain (FMRIB)'s software library (FSL 6.0.1)(Jenkinson et al., 2012), SPM12 (http://www.fil.ion.ucl.ac.uk) (Ashburner, 2012), and in-house MATLAB scripts (MATLAB 2018a, The MathWorks, Natick, MA). All images were manually reoriented to the axis of the anterior and posterior commissure. T1-weighted images were processed by the fsl_anat pipeline to generate the normalization matrix used to spatially normalize the pCASL data (Smith, 2004).

### 2.4 ^15^O-water PET perfusion quantification

Generating quantitative CBF images with PMRFlow requires determining whole-brain CBF, *f_wb_*, by PC MRI. The procedure involved drawing contours of the ICA and VA on the magnitude image that were copied to the phase image using in-house developed MATLAB scripts. Contours were visually inspected for correctness. Average velocity was calculated based on the linear relationship between phase change and velocity encoding (Nayak et al., 2015). Whole-brain CBF was quantified by multiplying the average velocity within each vessel by its cross-sectional area, scaling by brain tissue mass, and summing contributions from all vessels.

Perfusion images were generated using the following equation (Ssali et al., 2018):

(1) $$f_{i}=\frac{\int_{0}^{T}C_{i}\left(t\right)dt}{\frac{1}{f_{\mathrm{wb}}}\int_{0}^{T}C_{\mathrm{wb}}\left(t\right)dt+\frac{1}{\lambda }\int_{0}^{T}\int_{0}^{t}C_{\mathrm{wb}}\left(s\right)dsdt-\frac{1}{\lambda }\int_{0}^{T}\int_{0}^{t}C_{i}\left(s\right)dsdt}$$

where *f_i_* is CBF in the i^th^ voxel, C_i_(t) the corresponding tissue ^15^O-water time activity curve (TAC), C_wb_(t) the whole-brain TAC, λ the partition coefficient of water, and *T* the integration time (5 min). The resulting perfusion maps were spatially normalized to the MNI template using a non-linear image registration tool (FNIRT) (Jenkinson and Smith, 2001) and smoothed by a 6 mm gaussian filter. This resulted in an effective resolution of 8.8–9.4 mm for PET.

### 2.5 pCASL MRI perfusion quantification

SD-pCASL images were motion corrected and then pairwise subtracted. FL_TE-pCASL perfusion weighted images were generated by linear combination of the Hadamard-encoded images. All pCASL data were registered to their corresponding M0 image using SPM12. The remaining processing steps were implemented using FSL’s Oxford ASL toolbox. ENABLE (Shirzadi et al., 2018) was implemented to remove poor quality image volumes. Perfusion was quantified using a single compartment model including Bayesian inference to perform kinetic modeling and to spatially regularize the images (Chappell et al., 2009). For FL_TE-pCASL, the CBF images were generated using a kinetic model that incorporated transit time data. Model parameters were based on the guidelines of the ASL consensus paper (Alsop et al., 2015). Images were normalized to the MNI template using FNIRT (Jenkinson and Smith, 2001), smoothed by an 8 mm Gaussian filter (resulting in an effective resolution of 9.2 and 9.6 mm for SD-pCASL and FL_TE-pCASL respectively), and intensity normalized to whole-brain perfusion measured by PC MRI (i.e., whole-brain perfusion measured by ^15^O-water and pCASL were equivalent).

### 2.6 Statistics

Statistical analysis was performed using MATLAB and R (R Core Team 2013).

#### 2.6.1 Generating hypoperfusion maps using case control analysis

Recognizing the heterogeneity between FTD subtypes, perfusion images from each patient were compared individually to the groupwise perfusion images from the controls (Anazodo et al., 2018). Crawford and Howell’s modified *t*-test was used to account for the small sample of the control group. Treating the control mean and standard deviation as sample statistics, allowed for better characterization of the uncertainties in these values, thereby minimizing Type I errors (Crawford and Garthwaite, 2012). Although regional hyperperfusion has been reported in specific FTD subtypes (Dukart et al., 2010, Olm et al., 2016), this study only focused on evaluating regional hypoperfusion given that it is associated with cognitive impairment (Du et al., 2006). The critical t-value for a one-sided *t*-test was determined based on the size of the control group for alpha 0.05. Hypoperfusion maps for each patient were generated using absolute perfusion (aCBF) and relative perfusion (rCBF, intensity normalized to whole-brain CBF). For each technique, regional hypoperfusion identified by aCBF and rCBF were compared based on the change in the volume of the clusters and their location. Additionally, the percent change in cluster volumes between ^15^O-water relative to FL_TE-pCASL and SD-pCASL were evaluated.

#### 2.6.2 Agreement between PET and MRI-based hypoperfusion

Agreement of hypoperfusion detected by ^15^O-water and pCASL was characterized in terms of sensitivity and specificity. Twelve ROIs commonly associated with FTD and PSP and one reference region were selected from wfupickatlas: the amygdala, anterior cingulate cortex, inferior frontal gyrus, insula, midbrain, orbitofrontal gyrus, precuneus, supplementary motor area, superior frontal gyrus, temporal pole, middle temporal lobe, superior temporal lobe, and the occipital lobe (reference region). ROIs were classified as hypoperfused if the cluster size was greater than a sphere with diameter of 10 mm (i.e., 65 connected and significantly hypoperfused voxels) (*p* < 0.05). This threshold corresponds to the intrinsic resolution of both the PET and MRI perfusion images (i.e., 9.6 mm for PET and 9.2 mm for pCASL). This method is similar to visual rating scales where regional atrophy is identified within disease specific ROI (Harper et al., 2016). Agreement was calculated using the regions detected by ^15^O-water as the gold standard. Sensitivity represents the proportion of hypoperfused ROIs identified by ^15^O-water that were also identified as hypoperfused by pCASL, and specificity represents the proportion of ROIs with normal perfusion identified by both ^15^O-water and pCASL. It is important to distinguish that this is a measure of similarity between hypoperfusion maps generated by ^15^O-water and pCASL rather than an assessment of the clinical accuracy of regional hypoperfusion.

Voxel-by-voxel agreement between ^15^O-water and pCASL hypoperfusion maps was characterized based on the number of voxels that were overlapping (voxels detected by both pCASL and ^15^O-water), adjacent (voxels detected by pCASL that are adjoining overlapping voxels), and isolated (pCASL voxels not connected to clusters adjoining ^15^O-water regions). These parameters were expressed as a percent of the total number of hypoperfused voxels detected by pCASL. The Jaccard similarity coefficient was calculated to characterize the similarity between hypoperfusion detected by pCASL relative to ^15^O-water (Jaccard, 1912).

Paired t-tests, linear regression, and Bland-Altman plots were used to compare ROI-based perfusion measured by ^15^O-water and pCASL. Statistical significance was set to *p* < 0.05.

## 3 Results

### 3.1 Participants

Two patients were excluded due to issues with ^15^O-water production in one case and an unforeseen illness in the other case. One svFTD patient received an oral dose of lorazepam prior to the scan to manage anxiety associated with claustrophobia. This patient was excluded from the aCBF analysis due to its known effects on global CBF. FL_TE-pCASL data were acquired in nine control and all eight patients. Demographics and clinical characteristics of the participants are summarized in Table 1.

### 3.2 Whole-Brain perfusion

**Fig. 1:**
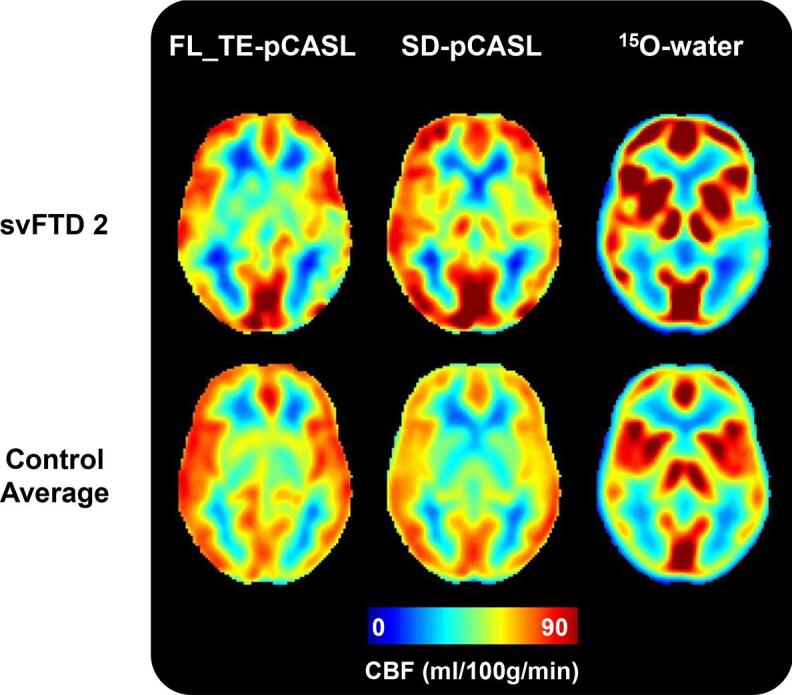
Perfusion maps generated by FL_TE-pCASL, SD-pCASL, and ^15^O-water from one patient participant (svFTD 2, top) and the average of all control participants (bottom). Perfusion maps are presented in radiological orientation.

Whole-brain CBF measured by PC MRI was 41 ± 8.6 and 48.1 ± 7.7 ml/100 g/min in patients and controls, respectively. Mean perfusion by FL_TE-pCASL, SD-pCASL, and PC MRI are summarized in Supplemental Table 1. To remove variability in perfusion due to differences between sequence parameters and imaging modalities, all perfusion data were intensity normalized to perfusion by PC MRI (Aslan et al., 2010). All perfusion maps showed the expected contrast of higher perfusion in grey matter relative to white matter (Fig. 1). Grey-to-white matter contrast by FL_TE-pCASL, SD-pCASL, and ^15^O-water across all participants were 1.5 ± 0.2, 1.5 ± 0.3 and 2.7 ± 0.3 respectively. There was no difference in grey-to-white matter contrast between patients and controls or between SD-pCASL and FL_TE-pCASL; however, ^15^O-water was significantly higher than both SD-pCASL and FL_TE-pCASL. Compared to ^15^O-water, the two pCASL sequences showed lower perfusion in the basal ganglia, and grey matter perfusion appeared more dispersed. Despite these differences, disease-related regional hypoperfusion was apparent in the CBF images from all three methods. For example, low perfusion was observed in the left middle temporal gyrus for the patient svFTD 2 (Fig. 1).

### 3.3 Regional hypoperfusion detected by PET and MRI

#### 3.3.1 Qualitative agreement of hypoperfusion maps

**Fig. 2:**
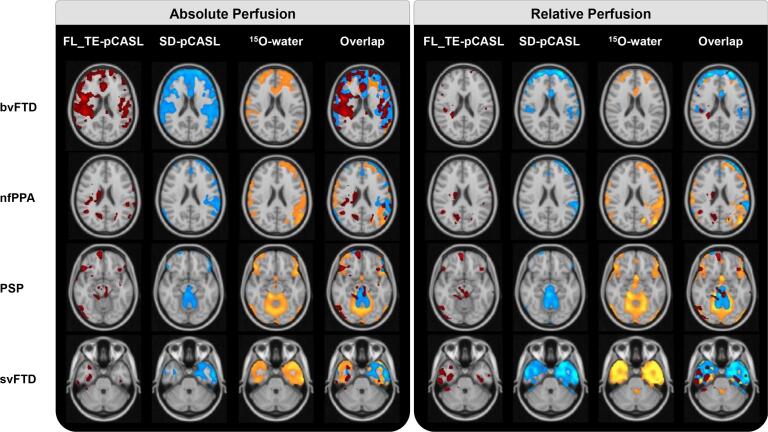
Regional hypoperfusion detected by FL_TE-pCASL, SD-pCASL, and ^15^O-water for one patient from each of the FTD subtypes/PSP. Images are in radiological orientation.

Example hypoperfusion maps detected by FL_TE-pCASL, SD-pCASL, and ^15^O-water for aCBF and rCBF are shown in Fig. 2. In patients with bvFTD (n = 2), all techniques detected hypoperfusion in the inferior and anterior temporal pole, superior and middle frontal gyrus, frontal pole and anterior cingulate (bilaterally for all regions). In addition, the pCASL sequences detected hypoperfusion in the insula bilaterally. For nfPPA (n = 2), all techniques identified left lateralized hypoperfusion in the temporal pole, inferior temporal gyrus, insula, frontal pole and perisylvian area. Inferior regions of the frontal pole showed bilateral hypoperfusion by ^15^O-water, whereas SD-pCASL primarily detected hypoperfusion on the left side. Only FL_TE-pCASL detected hypoperfusion in the right thalamus and caudate. For PSP (n = 2), hypoperfusion was detected in the midbrain, inferior frontal gyrus, and precuneus by all techniques. SD-pCASL and ^15^O-water also identified regional hypoperfusion in the anterior cingulate and superior frontal gyrus. Finally, asymmetric (left dominant) hypoperfusion in the temporal lobe (temporal pole, middle temporal gyrus, inferior temporal gyrus, and temporal fusiform cortex) was identified by all techniques in patients diagnosed with svFTD (n = 2). ^15^O-water and SD-pCASL identified hypoperfusion bilaterally, with greater hypoperfusion on the left, whereas FL_TE-pCASL only identified hypoperfusion on the right side.

The proportion of significantly hypoperfused voxels are summarized in Supplemental Table 2. Generally, similar regions of hypoperfusion were identified in the rCBF images and there was no difference in the volume of hypoperfusion clusters compared to aCBF. However, greater variability across patients was observed after normalisation. Specifically, ^15^O-water and SD-pCASL both showed a 3-to-14 fold increase in hypoperfused voxels in 3 patients (1 nfPPA, 2 svFTD), while the opposite, a 3-to-9 fold decrease, was found in 3 patients (2 bvFTD, 1 PSP) and minimal change (1.4 decrease to 1.1 fold increase) in the remaining two (1 nfPPA, 1 PSP). FL_TE-pCASL showed similar trends, except for a much greater increase for one svFTD patient.

Larger regions of hypoperfusion tended to be detected by PET; however, only cluster sizes detected by FL_TE-pCASL were significantly smaller. On average, SD-pCASL and FL_TE-pCASL detected 20.4 ± 38.2 and 21.3 ± 52.9% smaller volumes, respectively, compared to the aCBF from ^15^O-water and 8 ± 37.5 and 41.9 ± 40.6% smaller volumes, respectively, than the ^15^O-water rCBF images. However, 44% more hypoperfused voxels were detected in the SD-pCASL rCBF images for the two bvFTD patients compared to ^15^O-water.

#### 3.3.2 Quantitative agreement hypoperfusion maps

Good agreement between regional hypoperfusion detected by ^15^O-water and pCASL was found in terms of both aCBF and rCBF. This observation was confirmed by the sensitivity and specificity calculations for SD-pCASL; 70% and 78%, respectively, for aCBF and 73% and 74%, respectively, for rCBF. Regional hypoperfusion detected by ^15^O-water and FL_TE-pCASL using aCBF also showed good sensitivity (71%) and specificity (73%). However, the sensitivity decreased to 43% for rCBF while the specificity (71%) remained within a similar range.

The percentage of overlapping, adjacent and isolated voxels identified by SD-pCASL and FL_TE-pCASL relative to ^15^O-water are summarized in Table 2. SD-pCASL had a similar proportion of overlapping and adjacent voxels, whereas FL_TE-pCASL had a smaller portion of overlapping and greater fraction of adjacent voxels. The proportions identified by SD-pCASL with rCBF were not different from those identified with aCBF. With FL_TE-pCASL, intensity normalization resulted in an increase (*p* < 0.05) in adjacent voxels only. For most patients either an increase or small decrease in the percentage of common voxels was found after intensity normalization (ns) (Table 2). However, normalization caused a substantial decrease in the proportion of voxels common to PET and SD-pCASL for patients with bvFTD (41 and 74%), and one patient with PSP (36%). A similar trend was observed with FL_TE-pCASL. For both ASL techniques, similar results were found if the analysis was limited to grey matter. For example, the average fraction of overlapping, adjacent and isolated voxels for aCBF from SD-pCASL were 49.6 ± 25.6, 43.8 ± 24.8, and 6.6 ± 11.4, respectively.

**Table 2:** Summary of overlap analysis (expressed as a percent) and Jaccard similarity index of hypoperfusion detected by FL_TE-pCASL and SD-pCASL ^15^O-water PET. Comparison was conducted using absolute and relative CBF.

**Absolute Perfusion**								
	FL_TE-pCASL				SD-pCASL			
	Overlap	Adjacent	Isolated	Jaccard	Overlap	Adjacent	Isolated	Jaccard
bvFTD1	*32*	*68*	*0*	*0.18*	*32*	*68*	*0*	*0.20*
bvFTD2	*44*	*56*	*0*	*0.27*	*55*	*45*	*0*	*0.36*
nfPPA1	*21*	*78*	*1*	*0.08*	*44*	*54*	*3*	*0.21*
nfPPA2	*12*	*26*	*62*	*0.05*	*27*	*32*	*41*	*0.12*
PSP1	*19*	*78*	*3*	*0.10*	*23*	*76*	*0*	*0.14*
PSP2	*8*	*90*	*2*	*0.05*	*15*	*80*	*5*	*0.09*
svFTD1	*76*	*24*	*0*	*0.00*	*59*	*32*	*9*	*0.05*
svFTD2	*17*	*26*	*56*	*0.04*	*83*	*16*	*1*	*0.28*
svFTD3	*40*	*60*	*0*	*0.24*	*51*	*48*	*1*	*0.37*
**Mean ± SD**	*29.9 ± 21.3*	*56.2 ± 25.2*	*13.9 ± 25.6*	*0.11 ± 0.1*	*43.4 ± 21.3*	*50 ± 21.8*	*6.5 ± 13.2*	*0.2 ± 0.12*

**Relative Perfusion**								
	FL_TE-pCASL				SD-pCASL			
	Overlap	Adjacent	Isolated	Jaccard	Overlap	Adjacent	Isolated	Jaccard

bvFTD1	*10*	*86*	*4*	*0.04*	*19*	*75*	*6*	*0.12*
bvFTD2	*4*	*73*	*23*	*0.02*	*15*	*72*	*13*	*0.10*
nfPPA1	*22*	*75*	*2*	*0.06*	*43*	*56*	*1*	*0.21*
nfPPA2	*29*	*60*	*11*	*0.11*	*35*	*51*	*14*	*0.19*
PSP1	*22*	*77*	*2*	*0.09*	*25*	*74*	*0*	*0.15*
PSP2	*2*	*84*	*14*	*0.01*	*10*	*72*	*19*	*0.05*
svFTD1	*41*	*53*	*6*	*0.05*	*68*	*31*	*1*	*0.25*
svFTD2	*46*	*49*	*5*	*0.10*	*80*	*19*	*1*	*0.38*
svFTD3	*22*	*65*	*12*	*0.05*	*43*	*46*	*11*	*0.25*
**Mean ± SD**	*22 ± 15.3*	*69.3 ± 13*	*8.8 ± 7.1*	*0.06 ± 0.04*	*37.5 ± 24*	*55.2 ± 20.4*	*7.3 ± 7.1*	*0.19 ± 0.1*

**Fig. 3:**
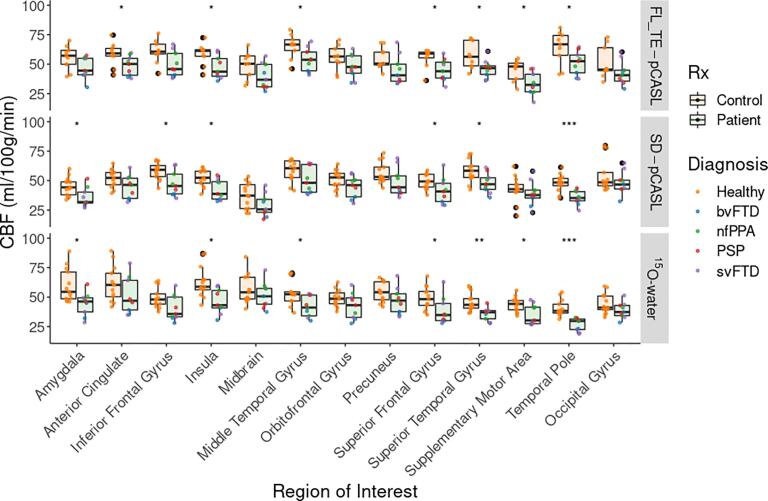
Perfusion measured by FL_TE-pCASL, SD-pCASL, and ^15^O-water in 12 FTD-specific ROIs and 1 reference ROI. Boxplots are grouped as patients (green) and controls (orange), and the colored points represent the diagnosis. Significance levels are denoted by: * (p < 0.05), ** (p < 0.001), *** (p < 0.0001).

**Fig. 4:**
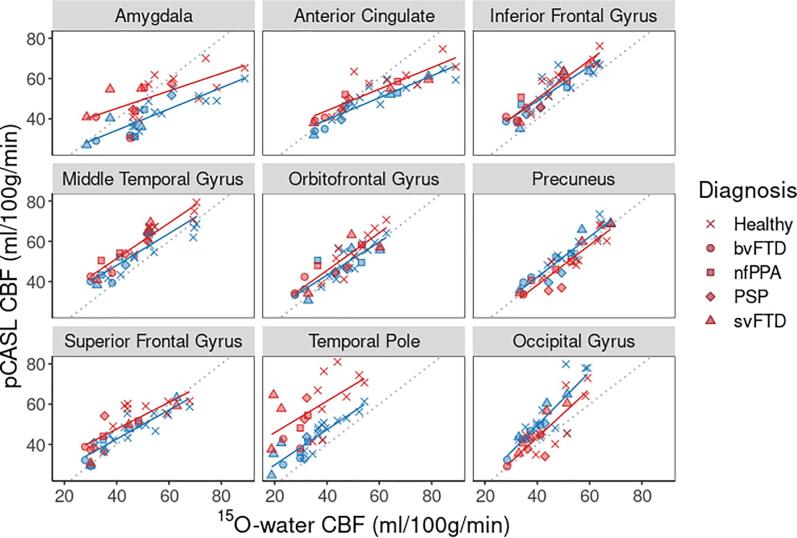
Comparison of ROI-averaged perfusion estimates from ^15^O-water and SD-pCASL (blue) and ^15^O-water and FL_TE-pCASL (red). Symbol shapes listed in the legend indicate FTD subtype. (For interpretation of the references to colour in this figure legend, the reader is referred to the web version of this article.)

**Fig. 5:**
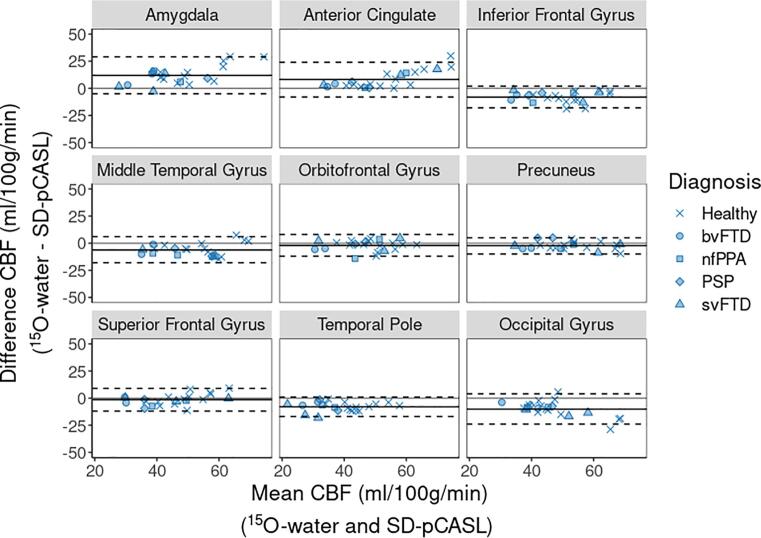
Bland-Altman plots show agreement between perfusion measured by SD-pCASL and ^15^O-water in ROIs common to FTD. Solid black line represents the average difference, and dashed black lines represent the 95% confidence interval.

Compared to controls, perfusion by ^15^O-water in patients was significantly lower in most FTD-specific ROIs (7 of 12) (Fig. 3). There was good agreement with SD-pCASL and FL_TE-pCASL; of the ROIs with significantly lower perfusion in patients, 5 were common to SD-pCASL and 6 to FL_TE-pCASL. All techniques showed no difference in perfusion between patients and controls in the occipital lobe, midbrain, and orbitofrontal gyrus. Perfusion measured by FL_TE-pCASL and SD-pCASL tracked well with ^15^O-water (Fig. 4) as indicated by the strong correlation (R > 0.75) in all ROIs, except for the supplementary motor area (Table 3). Additionally, most ROIs showed little proportional bias (Fig. 5, Supplemental Fig. 1).

**Table 3:** Linear regression intercept, slope and correlation coefficient for perfusion comparisons between (1) FL_TE-pCASL and ^15^O-water and (2) SD-pCASL and ^15^O-water in FTD-related ROIs and the occipital gyrus as a reference region.

	FL_TE-pCASL			SD-pCASL		
Region	Intercept	Slope	R	Intercept	Slope	R
Amygdala	28	0.43	0.66	13	0.53	0.84
Anterior Cingulate	23	0.53	0.87	18	0.54	0.91
Inferior Frontal Gyrus	12	0.96	0.93	15	0.86	0.88
Insula	20	0.59	0.86	18	0.55	0.86
Midbrain	−0.47	0.81	0.88	−5.4	0.72	0.78
Middle Temporal Gyrus	16	0.88	0.95	16	0.79	0.86
Orbitofrontal Gyrus	8.3	0.93	0.87	10	0.83	0.85
Precuneus	−1.3	0.99	0.91	2.2	1	0.93
Superior Frontal Gyrus	21	0.66	0.80	14	0.72	0.90
Superior Temporal Gyrus	7.3	1.1	0.90	6.9	1.1	0.93
Supplementary Motor Area	14	0.62	0.50	13	0.71	0.55
Temporal Pole	30	0.78	0.59	12	0.88	0.89
Occipital Gyrus	−4.6	1.2	0.82	−3.5	1.3	0.85

## 4 Discussion

This study assessed in FTD and related disorders the concordance of regional hypoperfusion identified by pCASL relative to PET using ^15^O-water, the gold standard for imaging perfusion. To appreciate the regional contrast of perfusion maps (Fig. 1), perfusion data were normalized to whole-brain CBF measured by PC MRI. Mean perfusion by PC MRI was 41 ± 8.6 ml/100 g/min across patients and 48.1 ± 7.7 ml/100 g/min in controls. The latter is consistent with previous reports involving older populations (Binnewijzend et al., 2014, Kilroy et al., 2014). SD-pCASL and ^15^O-water CBF maps had similar resemblance (Fig. 1), although greater grey-to-white matter contrast was observed in the PET images (2.7 ± 0.3) compared to either pCASL sequence (1.5 ± 0.2), similar to previous studies (Fan et al., 2016, Heijtel et al., 2014, Puig et al., 2019, Qiu et al., 2010, Zhang et al., 2014). This difference is likely related to the limitation of ASL to accurately measure white matter perfusion due to longer transit times (Van Gelderen et al., 2008, Zhang et al., 2014). Another difference was the higher CBF values obtained with ^15^O-water in sublobar regions such as the amygdala and insula. On average ^15^O-water values were 25 ± 17% higher than the corresponding SD-pCASL values (Fig. 1). ASL has been shown to underestimate perfusion in these regions (Fan et al., 2019, Heijtel et al., 2014), while PMRFlow can overestimate CBF due to neglecting blood volume signal contributions (Ssali et al., 2018). However, these discrepancies in absolute CBF between ^15^O-water and pCASL are less important for the case-control analysis since it only depends on changes in regional CBF. Similarly, a recent study reported no significant difference in CBF changes measured by pCASL and PET during an acetazolamide challenge despite discrepancies in absolute CBF (Puig et al., 2019). The strong correlation between ROI-based perfusion measured by SD-pCASL and ^15^O-water (R = 0.85 ± 0.1) demonstrated good agreement in the perfusion changes measured by the two methods (Fig. 4, Table 3). Furthermore, these results are within the range reported in a previous study that assessed agreement between ASL and ^15^O-water in a population of young healthy participants (R = 0.61–0.87) (Zhang et al., 2014).

Both ^15^O-water and SD-pCASL detected hypoperfusion in regions previously shown to have hypometabolism (Fig. 2) (Diehl-Schmid et al., 2007, Foster et al., 1988, Moodley et al., 2015, Teune et al., 2010). Similar to previous FDG PET studies (Anazodo et al., 2015, Ceccarini et al., 2020), the extent and intensity of clusters detected by ^15^O-water tended to be larger – on average SD-pCASL detected a 20 ± 38% smaller volume of hypoperfusion – suggesting PET had greater sensitivity. Anatomical regions associated with the overlapping clusters’ coordinates were in regions known to be associated with each FTD subtype. Ninety-three percent of hypoperfused voxels identified by SD-pCASL were either overlapping with (43%) or adjacent to (50%) voxels identified by ^15^O-water, and less than 7% of voxels were found in isolated clusters. This is in agreement with the sensitivity/specificity analysis: 70% of hypoperfused ROIs and 78% of ROIs with normal perfusion identified by SD-pCASL were common to ^15^O-water. In contrast, the Jaccard similarity index, which is a commonly used imaging metric, did not adequately capture the similarities (Table 2). This discrepancy is likely related to the large proportion of adjacent, rather than overlapping, voxels when comparing hypoperfusion maps generated by ^15^O-water and SD-pCASL. Even for the svFTD patient shown in Fig. 2, in which there was an 83% overlap between ^15^O-water and pCASL clusters, the Jaccard index was 0.28 (Table 2).

Unexpectedly, normalizing by whole-brain CBF caused considerable variability in terms cluster size of detected regional hypoperfusion (Fig. 2). Some patients showed the expected increase (e.g. svFTD) or minimal change (e.g. PSP and nfPPA), while others showed a decrease (e.g. bvFTD, PSP). This last group highlights a potential challenge associated with assessing relative perfusion changes. Intensity normalization is intended to remove between-subject variations, thereby allowing for a more sensitive assessment of regional hypoperfusion. However, normalizing by whole-brain CBF can diminish sensitivity to regional perfusion deficits if whole-brain CBF is significantly reduced by widespread disease effects (Borghammer et al., 2008). This is illustrated by the bvFTD patients for whom approximately 30% of the whole brain was significantly hypoperfused (Supplemental Table 2). Other reference regions were investigated for the bvFTD patients (specifically the occipital lobe and cerebellum), but the rCBF hypoperfusion maps remained relatively sparse (data not presented). These results highlight the benefit of quantitative imaging for assessing regional hypoperfusion. That is, it is valuable to compare regional hypoperfusion patterns identified by aCBF and rCBF images to assess if perfusion normalization results in a substantial reduction in regional hypoperfusion, as evident with the bvFTD patients in this study.

FL_TE-pCASL also showed good agreement with ^15^O-water (sensitivity = 71% and specificity = 73%). In addition, there was good correlation between ROI-based perfusion estimates from the two methods (R = 0.81 ± 0.14) (Table 3). Unlike SD-pCASL, perfusion in the amygdala and insula ROIs were not significantly lower than the CBF estimates from ^15^O-water, demonstrating the added value of accounting for transit times. Despite these findings, the overlap between ^15^O-water and FL_TE-pCASL hypoperfusion maps had 30% fewer voxels compared to overlap between ^15^O-water and SD-pCASL (Table 2). In addition, intensity normalization reduced the sensitivity by roughly a half due to the decrease in the already small cluster sizes. Although FL_TE-pCASL was shown to have good sensitivity for detecting perfusion changes related to Moya-Moya disease (Fan et al., 2019), the hypoperfusion clusters detected in the current study appeared sparce and with poorer overlap with ^15^O-water maps (Fig. 2). One explanation is that FL_TE-pCASL is more sensitive to motion since data since 8 encoding steps are required to generate a set of difference images. In contrast, SD-pCASL only requires one pair of label and tag images. In this study, the shorter acquisition time for FL_TE-pCASL (roughly 5 min) likely contributed to its lower sensitivity compared to SD-pCASL, which had a 10-min acquisition time. In a companion study involving the same cohort, we found that while a PLD of 2 s was sufficient to minimize the effects of transit time errors, 5 min of scanning was not adequate to accurately measure perfusion (Ssali et al., 2021). Nevertheless, the good correlation with ^15^O-water in terms of ROI-based perfusion (Fig. 4, Supplemental Fig. 1) shows the promise of FL_TE-pCASL.

While the results of this study highlight the promise of pCASL for detecting regional hypoperfusion related to FTD subtypes, there are a few limitations. First, data were acquired from a small sample, with only 2–3 participants per subtype. Despite the small sample size, there was good consistency in terms of regional hypoperfusion identified within each subtype by PET and MRI. Second, the acquisition time for SD-pCASL was closer to 10 min, rather than the recommended 5 min (Alsop et al., 2015). A longer scan time was chosen to improve the signal-to-noise ratio; however, it increases the risk of motion artefacts. The use of a labeling sequence that included background suppression and careful attention to minimizing head motion during imaging were implemented to minimize potential motion. Finally, partial volume correction was not applied to the perfusion images as this step is not commonly used in clinical practice. Although brain atrophy likely contributed to the hypoperfusion detected by PET and pCASL, a previous study reported similar perfusion differences between dementia patients and controls with and without partial volume correction (Binnewijzend et al., 2014).

In conclusion, the present study demonstrates the potential of pCASL for assessing regional hypoperfusion related to FTD subtypes and PSP. Direct comparison of MRI and PET perfusion revealed that although ^15^O-water showed greater sensitivity, as indicted by larger clusters, SD-pCASL and FL_TE-pCASL identified hypoperfusion in similar regions, with the former showing strong agreement with the ^15^O-water results. Although rCBF and aCBF showed no significant differences in terms of spatial overlap and metrics of agreement with PET, rCBF showed considerable variability across subtypes, indicating that care must take when selecting a reference region. These results support the use of pCASL as a cost-effective alternative to PET for assessing regional perfusion deficits associated with FTD.

### CRediT authorship contribution statement

**Tracy Ssali:** Conceptualization, Data curation, Formal analysis, Investigation, Methodology, Project administration, Resources, Software, Validation, Visualization, Writing – original draft, Writing – review & editing. **Lucas Narciso:** Investigation, Methodology, Writing – review & editing. **Justin Hicks:** Investigation, Resources, Writing – review & editing. **Linshan Liu:** Investigation, Writing – review & editing. **Sarah Jesso:** Investigation, Resources, Writing – review & editing. **Lauryn Richardson:** Investigation, Resources, Writing – review & editing. **Matthias Günther:** Resources, Software, Writing – review & editing. **Simon Konstandin:** Resources, Software, Writing – review & editing. **Klaus Eickel:** Resources, Software, Writing – review & editing. **Frank Prato:** Supervision, Writing – review & editing. **Udunna C. Anazodo:** Investigation, Methodology, Resources, Writing – review & editing. **Elizabeth Finger:** Resources, Supervision, Writing – review & editing. **Keith St Lawrence:** Conceptualization, Formal analysis, Funding acquisition, Methodology, Project administration, Resources, Supervision, Validation, Writing – review & editing.

## Declaration of Competing Interest

The authors have no conflicts to declare.
