# Supplementary material for: Concordance of regional hypoperfusion by pCASL MRI and 15O-water PET in frontotemporal dementia: Is pCASL an efficacious alternative?
Source: Neuroimage Clin. 2022 Jan 31;33:102950. doi: 10.1016/j.nicl.2022.102950 (PMC8829802; doi:10.1016/j.nicl.2022.102950)
Supplement: Supplementary data 1 [file mmc1.docx]

# Concordance of Regional Hypoperfusion by pCASL MRI and ^15^O-water PET in Frontotemporal Dementia: Is pCASL an Efficacious Alternative?

**Author List**

| *Tracy Ssali, MSc | 1. Lawson Health Research Institute, London, Canada  2. Department of Medical Biophysics, Western University, London, Canada |
| --- | --- |
| Lucas Narciso, MSc | 1. Lawson Health Research Institute, London, Canada  2. Department of Medical Biophysics, Western University, London, Canada |
| Justin Hicks, PhD | 1. Lawson Health Research Institute, London, Canada  2. Department of Medical Biophysics, Western University, London, Canada |
| Linshan Liu, MSc | 1. Lawson Health Research Institute, London, Canada  2. Department of Medical Biophysics, Western University, London, Canada |
| Sarah Jesso | 1. Lawson Health Research Institute, London, Canada  3. St. Joseph’s Health Care, London, Canada |
| Lauryn Richardson | 1. Lawson Health Research Institute, London, Canada  3. St. Joseph’s Health Care, London, Canada |
| Matthias Günther, PhD | 4. Fraunhofer Institute for Medical Image Computing MEVIS, Bremen, Germany  5. University Bremen, Bremen, Germany |
| Simon Konstandin, PhD | 4. Fraunhofer Institute for Medical Image Computing MEVIS, Bremen, Germany  6. Mediri GmbH, Heidelberg, Germany |
| Klaus Eickel, PhD | 6. Mediri GmbH, Heidelberg, Germany |
| Frank Prato, PhD | 1. Lawson Health Research Institute, London, Canada  2. Department of Medical Biophysics, Western University, London, Canada |
| Udunna C Anazodo, PhD | 1. Lawson Health Research Institute, London, Canada  2. Department of Medical Biophysics, Western University, London, Canada |
| Elizabeth Finger, MD | 1. Lawson Health Research Institute, London, Canada  2. Department of Medical Biophysics, Western University, London, Canada  7. Department of Clinical Neurological Sciences, Western University, London, Canada |
| Keith St Lawrence, PhD | 1. Lawson Health Research Institute, London, Canada  2. Department of Medical Biophysics, Western University, London, Canada |

***Corresponding author:** Tracy Ssali, Street address: Lawson Health Research Institute, 268 Grosvenor St, London, Ontario, N6A4V2, Canada. Telephone: 519-646-6100 x 65732. Electronic mail: [tssali@uwo.ca](mailto:tssali@uwo.ca)

# Supplementary Material

## Supplemental Table 1

Supplemental Table 1: Whole-brain perfusion (in ml/100g/min) by FL_TE-pCASL, SD-pCASL, and phase contrast in patients and controls.

|  | Control CBF | Patient CBF |
| --- | --- | --- |
| FL_TE-pCASL | 85.8 ± 18.5 | 80.2 ± 15.9 |
| SD-pCASL | 60.8 ± 11.2 | 54.5 ± 10.2 |
| Phase Contrast | 48.1 ± 7.7 | 41 ± 8.6 |

## Supplemental Table 2

Supplemental Table 2: Percent of significantly hypoperfused voxels (with respect to whole brain) identified in each patient using FL_TE-pCASL, SD-pCASL, and ^15^O-water using aCBF and rCBF.

|  | FL_TE-pCASL | | SD-pCASL | | ^15^O-water | |
| --- | --- | --- | --- | --- | --- | --- |
|  | aCBF | rCBF | aCBF | rCBF | aCBF | rCBF |
| bvFTD1 | 20.3 | 3.0 | 23.0 | 6.5 | 22.1 | 5.3 |
| bvFTD2 | 37.5 | 5.5 | 35.6 | 7.0 | 39.0 | 4.2 |
| nfPPA1 | 7.1 | 5.0 | 9.1 | 9.5 | 13.3 | 14.0 |
| nfPPA2 | 1.1 | 2.7 | 1.0 | 4.1 | 1.5 | 4.9 |
| PSP1 | 11.4 | 8.4 | 14.2 | 14.5 | 12.9 | 14.2 |
| PSP2 | 14.8 | 3.3 | 9.5 | 2.9 | 7.9 | 2.8 |
| svFTD1 | 0.0 | 2.2 | 0.5 | 6.8 | 5.6 | 16.1 |
| svFTD2 | 1.5 | 4.4 | 2.1 | 9.1 | 5.9 | 17.2 |
| svFTD3 | - | 2.8 | - | 8.3 | - | 9.4 |

## Supplemental Figure 1


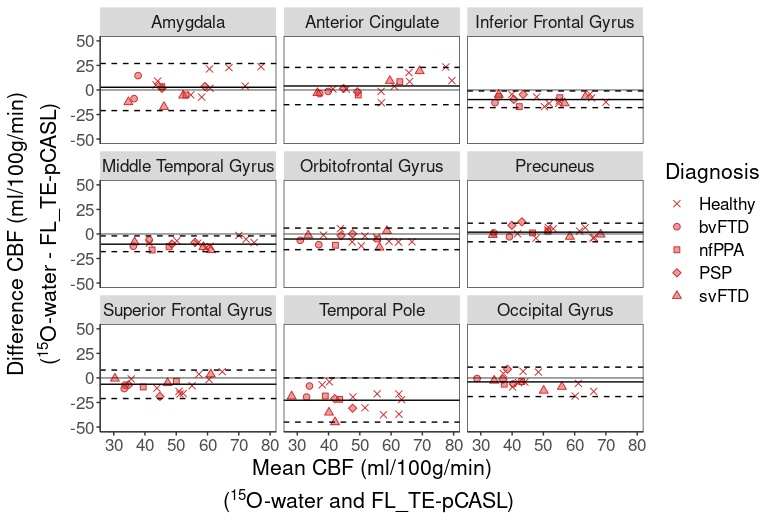


Supplemental Figure 1: Bland-Altman plots showing the agreement between perfusion measured by FL_TE-pCASL and ^15^O-water in specific ROIs. The solid black line represents the average difference, and dashed black lines represent the 95% confidence interval.
